# Divergent Gene Activation in Peripheral Blood and Tissues of Patients with Rheumatoid Arthritis, Psoriatic Arthritis and Psoriasis following Infliximab Therapy

**DOI:** 10.1371/journal.pone.0110657

**Published:** 2014-10-21

**Authors:** Alexander Rosenberg, Hongtao Fan, Yahui G. Chiu, Rebecca Bolce, Darren Tabechian, Rick Barrett, Sharon Moorehead, Frédéric Baribaud, Hao Liu, Nancy Peffer, David Shealy, Edward M. Schwarz, Christopher T. Ritchlin

**Affiliations:** 1 Division of Allergy, Immunology and Rheumatology, University of Rochester Medical Center, Rochester, NY, United States of America; 2 The Center for Musculoskeletal Research, University of Rochester Medical Center, Rochester, NY, United States of America; 3 Janssen Research and Development LLC, Spring House, PA, United States of America; University of Leuven, Rega Institute, Belgium

## Abstract

**Objective:**

The immune inflammatory disorders rheumatoid arthritis (RA), psoriatic arthritis (PsA) and psoriasis (Ps) share common pathologic features and show responsiveness to anti-tumor necrosis factor (TNF) agents yet they are phenotypically distinct. The aim of this study was to examine if anti-TNF therapy is associated with divergent gene expression profiles in circulating cells and target tissues of patients with these diseases.

**Methods:**

Peripheral blood CD14^+^ and CD14^−^ cells were isolated from 9 RA, 12 PsA and 10 Ps patients before and after infliximab (IFX) treatment. Paired synovial (n = 3, RA, PsA) and skin biopsies (n = 5, Ps) were also collected. Gene expression was analyzed by microarrays.

**Results:**

26 out of 31 subjects responded to IFX. The transcriptional response of CD14^+^ cells to IFX was unique for the three diseases, with little overlap (<25%) in significantly changed gene lists (with PsA having the largest number of changed genes). In Ps, altered gene expression was more pronounced in lesional skin (relative to paired, healthy skin) compared to blood (relative to healthy controls). Marked suppression of up-regulated genes in affected skin was noted 2 weeks after therapy but the expression patterns differed from uninvolved skin. Divergent patterns of expression were noted between the blood cells and skin or synovial tissues in individual patients. Functions that promote cell differentiation, proliferation and apoptosis in all three diseases were enriched. RA was enriched in functions in CD14^−^ cells, PsA in CD14^+^ cells and Ps in both CD14^+^ and CD14^−^ cells, however, the specific functions showed little overlap in the 3 disorders.

**Conclusion:**

Divergent patterns of altered gene expression are observed in RA, PsA and Ps patients in blood cells and target organs in IFX responders. Differential gene expression profiles in the blood do not correlate with those in target organs.

## Introduction

Immune mediated inflammatory disorders are a group of diseases that share several common features including pathologic mechanisms characterized by proliferation and accumulation of immune cells, increased release of TNF and other cytokines and altered tissue remodeling. Other common features include cardiovascular and metabolic comorbidities and responsiveness to anti-Tumor Necrosis Factor (TNF) agents [1]–[3]. While TNF blockade has proven to be a highly effective treatment for rheumatoid arthritis (RA), psoriatic arthritis (PsA) and psoriasis (Ps), three of the most prevalent immune mediated inflammatory disorders, recent evidence indicate that each disease arises by distinct pathophysiologic mechanisms. For example, RA is strongly linked to MHC class II genes and citrullinated autoantibodies with pathogenic potential whereas PsA and Ps share strong MHC Class I associations and disease-specific antibodies have not been identified [4]–[6]. From a therapeutic perspective, agents that target B and T cells are highly effective in RA [7] but not in PsA or Ps [8], [9], and methotrexate, a cornerstone drug in RA and Ps, is not effective in PsA [8]–[10]. Lastly, molecules in the IL-23/Th17 pathway are important targets in Ps [11], [12] and PsA [13] but do not show great promise in RA [14].

A central question that remains to be addressed is whether TNF inhibition has divergent effects on key gene networks in these three diseases. Over the past decade, investigators have turned to microarray analytic techniques of peripheral blood cells and target tissues (synovium, skin) to examine cross-sectional (compared to control samples) and longitudinal (before and after therapy) gene expression [15]. From these studies, several fundamental insights emerged. First, the molecular network in the immune mediated inflammatory disorders is far more complex than expected [16]. Second, cross-sectional differential gene expression is much lower in blood cells and in specific cell lineages compared to whole tissues [15]. Third, gene expression signatures in blood cells and synovial biopsies are heterogeneous and very patient-specific [17]. Fourth, to date, no pre-treatment gene expression profile in blood or tissue can accurately and reliably predict response to anti-TNF therapy in any of these three diseases [18]–[20]. Despite these caveats, microarray studies in autoimmune disorders (multiple sclerosis, SLE, Crohn’s disease, ulcerative colitis, juvenile rheumatoid arthritis and type 1 diabetes) reveal shared perturbations of common cellular processes, particularly apoptosis, regulation of cytokines and T cell activation [21].

Taken together, microarray studies reveal a complex, heterogeneous immune inflammatory response in the immune mediated inflammatory diseases yet common signatures, as outlined above, are characteristic of specific autoimmune diseases. Given the marked effects of TNF inhibition on patient reported outcomes, systemic inflammation and tissue remodeling in RA, PsA and Ps, genomic analysis of cells and tissues before and after treatment has the potential to unveil pivotal overlapping and disease-specific transcriptional events in disease pathogenesis. We hypothesize that TNF inhibition will result in differential effects on gene expression in the blood cells and target tissues that will be specific to each disorder-RA, PsA and Ps. To examine this hypothesis, we analyzed cells associated with innate immunity (CD14^+^ monocytes) and cells primarily associated with acquired immune responses (CD14^−^ T and B lymphocytes) separately and compared gene expression in the blood to profiles observed in target tissues (synovium, skin) before and after IFX therapy.

## Patients and Methods

### Patients

**Figure 1:**
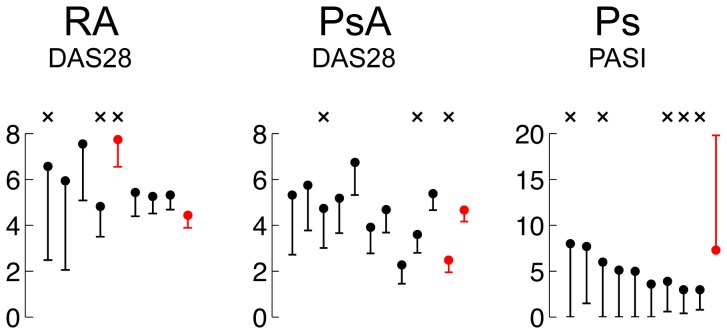
Disease activity before and after IFX for all RA, PsA and Ps subjects in study. Closed circles and horizontal line segments represent disease activity at baseline and 10 weeks post IFX, respectively. For RA and PsA, x’s above the plots indicate patients that donated knee biopsy at baseline and 10 weeks post IFX. For Ps, they indicate patients that donated lesional and non-lesional skin at baseline as well as lesional skin 2 weeks post IFX. Red indicates IFX non-responders. For RA and PsA, a non-responder had an improvement in DAS28 of less than 1.2 (if the endpoint DAS28>5.1) or less than 0.6 (if the endpoint DAS28<5.1). For Ps, a non-responder had <50% reduction in PASI due to the treatment.

Between April 2007 and June 2009, 31 patients with active RA, PsA and Ps who were naïve to anti-TNF agents, were recruited from the Faculty Rheumatology Clinics at the University of Rochester Medical Center after informed, written consent was obtained in a protocol approved by the Research Subjects Review Board at the University of Rochester Medical Center. Of the 31 subjects, 9 had active RA [22] and 12 had PsA [23] despite treatment with Disease Modifying Anti-Rheumatic Drugs (DMARDs). Also, 10 patients with extensive Ps (>5% Body Surface Area, BSA) documented by a dermatologist, were enrolled and they were examined by a rheumatologist to exclude the presence of inflammatory arthritis (see Table 1 and Figure 1). Nineteen healthy controls were also recruited.

**Table 1:** Patient demographics.

	RA	PsA	Ps	HC
n	9	12	10	19
Age (mean ± s.d.)	51.6±10.9	52.7±13.3	40.9±15.1	42±13.3
Gender (m/f)	1/8	6/6	4/6	6/13
Dx Duration (mean ± s.d.)	9.8±5.7	8.1±6.0	10.1±7.0	
Baseline Methotrexate	6	8	0	
Baseline Hydroxychloroquine	1	0	0	
Baseline Leflunomide	1	0	0	
Baseline topicals	0	0	10	
No Baseline Meds	1	4	0	
Activity Measure	DAS28	DAS28	PASI	
Baseline Activity (mean ± s.d.)	6.03±1.23	4.54±1.73	5.26±1.92	

A Disease Activity-C Reactive Protein (DAS-CRP) score was calculated based on a 28-joint count performed on the RA and PsA patients at baseline, week 2 and week 10 [24] and a Psoriasis Area and Severity Index (PASI) score [25] was obtained on the Ps subjects at the same time points; baseline and 10 week scores are shown in Figure 1. Patients were considered non-responders if the improvement in DAS28<1.2 (if the endpoint DAS28>5.1) or if the improvement in DAS28<0.6 (if the endpoint DAS28<5.1). In a similar fashion, we used the PASI50 (<50% reduction in PASI) to determine non-responders in our Ps subjects.

### Skin and synovial biopsies

4 mm punch biopsies were performed on involved and uninvolved skin at baseline in 5 Ps patients. A repeat biopsy was performed at week 2 after IFX therapy at a site adjacent to the baseline biopsy of involved skin. Synovial biopsies were performed on the knee of 3 RA and 3 PsA subjects with a Parker Pearson biopsy needle (Dyna Medical, London, Canada) under ultrasound guidance at baseline and repeated on the same knee at week 10. Several synovial biopsy specimens (6–10) were retrieved in each knee from areas of inflamed synovium and the samples were pooled for transcriptome analysis from each patient.

### Blood Sampling for RNA isolation

20 mL of blood was collected from registered subjects at baseline, week 2 and week 10 and purified by ficol gradient. CD14^+^ cells were positively selected by Miltenyi, anti-CD14 magnetic beads following manufacturer’s instructions. Briefly, ficol-purified PBMC were incubated with anti-CD14 beads (Miltenyi Biotech, Cambridge, Mass) and added into a Miltenyi column under the field of magnetic force. The unbound cells (CD14^−^) were collected and considered as flow through. Bound CD14^+^ cells were flushed out and purity (>90%) was checked by flow cytometry. Additionally, CD14 gene expression assessed by microarrays was consistently high (∼12 in log_2_ units) in every patient sample derived from CD14^+^ cells.

### RNA Isolation and quantification

Total RNA was isolated from CD14^+^ monocytes and CD14^−^ cells using the RNeasy Mini Kit with DNase digestion on the column (Qiagen, Inc. Valencia, CA, USA) as per the manufacturer’s instructions. Total RNA was isolated from biopsy samples using the RNeasy Lipid Tissue Mini Kit with DNase digestion on the column (Qiagen, Inc. Valencia, CA, USA) as per the manufacturer’s instructions. The quality and quantity of RNA was assessed using the Agilent 2100 Bioanalyzer (South Plainfield, NJ, USA) or the Caliper LabChip GX (PerkinElmer, Inc. Waltham, MA, USA). Only those RNAs with a RNA Integrity Number (RIN, Agilent) or RNA Quality Score (RQS, PerkinElmer) of greater than 4.0 (out of 10) were submitted for microarray. CD14^+^ depleted PBMC and CD14^+^ monocyte RNAs had an average RQS of 9.4, and biopsy RNAs had an average RQS/RIN of 6.7.

### Microarray data analysis

RNA samples were processed using the High Throughput HG133plus2 PM microarray platform (Affymetrix Inc., Santa Clara CA) at the Janssen R&D microarray core facility in La Jolla, CA. Arrays were processed in an order random with respect to sample groups in order to minimize the intrusion of batch effects. The CEL files were normalized using RMA algorithm implemented in ArrayStudio version 4.1 (OmicSoft, Durham, NC.). The MAS5 QC report and principal component analysis (PCA) were used for outlier detection before proceeding to analysis. A General Linear Model (GLM) implemented in ArrayStudio was used to detect significantly differentially expressed genes. Significant genes were based on the following 3 criteria: 1. A fold change in the comparison greater than 1.5; 2. A raw P value less than 0.05; 3. A mean expression value equal or higher than 5 (log_2_ value) in either control or disease samples. For biopsy specimens, differential gene expression was analyzed using paired t-tests and genes were selected based on p-value (affymetrix probe sets with no associated gene annotation were not included). For heat maps, when multiple probe sets for a single gene were available in statistical lists, the one with the lowest p value was chosen as a representative for visualization. Gene list relationships were analyzed using hive plots. Ingenuity Pathway Analysis (IPA, Ingenuity Systems, Redwood City, CA) was used to find enriched annotations and predicted transcription factors [26]. Clustering, additional statistical tests and visualizations were performed using Matlab (MathWorks, Natick, MA). The microarray data discussed in this publication have been deposited in NCBI’s Gene Expression Omnibus [27]and are available through GEO Series accession number GSE57386 (http://www.ncbi.nlm.nih.gov/geo/query/acc.cgi?acc=GSE57386).

### RT-PCR validation of microarray data

Validation of microarray results from cells and biopsy samples by real-time polymerase chain reaction (RT-PCR) was performed on a subset of genes that met significance criteria described above, and were of biological interest. RNA from CD14^+^ and CD14^−^ cells was reverse transcribed using the High-capacity cDNA Reverse Transcription kit (Life Technologies, Grand Island, NY). RNA from biopsies was amplified using the Ovation RNA Amplification System V2 (NuGen Technologies, San Carlos, CA) RT-PCR reactions were performed using TaqMan Gene Expression Assays (Life Technologies, Grand Island, NY) and run on an Applied Biosystems ViiA7 instrument under standard cycling conditions. TaqMan Gene Expression Assays are listed in Tables S2–S5. Statistical analysis of RT-PCR data was conducted using Prism version 6.05 (GraphPad Software, La Jolla CA) and ArrayStudio. TaqMan data (after normalization to GAPDH), was first log2 transformed and paired T test (if paired samples) was used to determine the significance and fold change of gene expression change; when there were no paired samples, GLM (General Linear Model) implemented in ArrayStudio was used to conduct the statistical tests.

## Results

### Patient Characteristics

The demographics and baseline medications for the patients in the 3 groups are outlined in Table 1. The Ps patients were younger than the RA or PsA patients and the RA patients were almost all female. The average disease duration ranged between 8 and 10 years and most of the patients with RA and PsA were inadequate responders to disease modifying anti-rheumatic agents (DMARDs) and the most commonly prescribed medication was methotrexate. Eight of the 9 RA patients were positive for anti-CCP antibodies. Patients had active skin and joint disease as measured by the Psoriasis Area and Severity Index (PASI) and BSA (>5%), and Disease DAS28-CRP scores, respectively.

### Treatment Response

The baseline DAS28-CRP scores in RA and PsA were 6.03 and 4.54 and the baseline PASI score in the Ps subjects was 5.26. The mean change following IFX therapy in the DAS28 in RA was 1.90 (p<.007), in PsA was 1.19, (p<.001) and the mean change in PASI for the nine responders was 4.6±1.8 (p<.008). The DAS28 and PASI scores for individual subjects are depicted in Figure 1. All patients in the RA and PsA groups had lower DAS28 scores at week 10. One Ps patient had an increase in the PASI score at week 10. In all other Ps patients, the scores improved.

### General transcriptomic changes

In general, transcriptomic changes in the peripheral blood subsets for all diseases were less striking than those found in Ps biopsy samples, and did not maintain their significance when accounting for multiple comparisons. Tables 2 and 3 summarize the results of various group comparisons. In Table 2, numbers of significant genes found within each cell type for cross-sectional comparisons with healthy controls, or longitudinal comparisons to baseline are shown. Note that for all diseases, for both CD14^+^ and CD14^−^, greater transcriptomic changes were observed in the baseline disease samples relative to healthy controls than in the treated disease samples relative to baseline disease, with the exception of PsA at 10 weeks (see Figures S1 and S2 for cross-sectional disease comparisons). For the treatment effect comparisons, the RA samples revealed a similar degree of changes at both time points, whereas in PsA and Ps, most of the significant gene changes were at the 10 week time point for CD14^+^ cells. For CD14^−^, RA had more changes at 2 weeks whereas PsA and Ps had more changes at 10 weeks. The results for the biopsy comparisons are shown in Table 3. The most dramatic response was seen in Ps with the most significant genes found when comparing non-lesional and psoriatic skin from the same patients. Far fewer significant genes were found in the knee biopsy samples but the sample size for those comparisons was very small.

**Table 2:** Numbers of significant genes in PBMC comparisons.

		RA (n = 9)			PsA (n = 12)			Ps (n = 10)		
Comparison	Cell	Total	Up	Down	Total	Up	Down	Total	Up	Down
Disease (Dx vs. HC at BL)	CD14^+^	349	170	179	81	14	67	125	76	49
	CD14^−^	371	259	112	197	53	144	441	184	257
Treatment (2 wk vs. BL)	CD14^+^	77	42	35	75	62	13	29	15	14
	CD14^−^	141	90	51	4	4	0	9	6	3
Treatment (10 wk vs. BL)	CD14^+^	69	56	13	140	71	69	102	85	17
	CD14^−^	76	32	44	225	53	172	67	59	8

Numbers reflect total number of genes as well as the number up- and down-regulated. These numbers were derived from a slightly larger number of probe sets. Unannotated probe sets are not included in this tabulation. BL = baseline, HC = healthy control (n = 19), Dx = disease. Numbers are based on general linear model with p<.05 and fold change at least 1.5.

**Table 3:** Numbers of significant genes in biopsy comparisons.

	RA (n = 3)			PsA (n = 3)			Ps (n = 5)		
Comparison	Total	Up	Down	Total	Up	Down	Total	Up	Down
L skin at BL vs. NL skin at BL	−	−	−	−	−	−	1417	850	567
L tissue at 2 or 10 wk vs. L tissue at BL	64	32	32	52	26	26	326	74	252
L skin at 2 wk vs. NL skin at BL	−	−	−	−	−	−	203	102	101

Numbers reflect total number of genes as well as the number up- and down-regulated. These numbers were derived from a slightly larger number of probe sets. Unannotated probe sets are not included in this tabulation. BL = baseline, L = lesional, NL = non-lesional. Numbers are based on paired t-test with p<.01 and fold change at least 1.5.

### Validation of gene expression

Genes with significant changes in expression and of biological interest were selected for validation with RT-PCR assays and are shown in Tables S2–S4. The correlation between expression on microarray and RT-PCR for the CD14^+^ cells was 100% (fold-change in same direction) or 67% (fold-change in same direction and significant p ≤ 0.05) based on the group comparisons. The agreement between expression change by microarray and by RT-PCR for the biopsies was 97% in terms of direction of change. In addition to having the same direction of change, 78% were also significant by both measures (p ≤ 0.05). Furthermore, we compared the top 50 genes for paired lesional vs. non-lesional Ps samples in a recent meta-study [28] to our data (a subset of microarrays in this study) and found remarkable consistency (see Figure S5), indicating that the microarray measurements we report are capable of reproducing published results.

### IFX response in CD14^+^ cells

**Figure 2:**
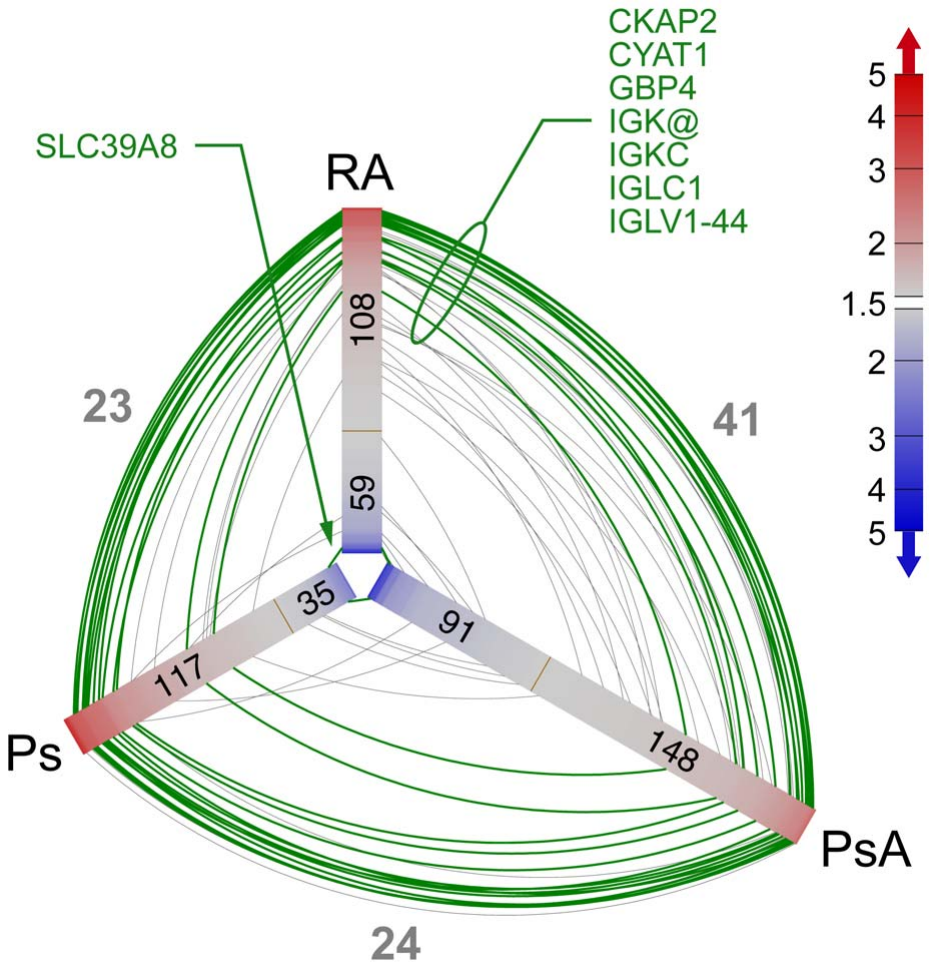
Venn/hive plots showing genes that changed following IFX treatment in three diseases in CD14^+^ cells. The three shaded bars emanating from the center each represent a list of significant probe sets where their members are distributed along the bar sorted by fold-change (indicated by color). The numbers of probe sets up- and down-regulated are shown on the red and blue portions of the bars, respectively. For example, in PsA,148 genes were up-regulated and 91 down-regulated following IFX therapy. Arcs that connect pairs of shaded bars represent genes that are common between two comparisons (in the same fashion, the intersect of two circles in a Venn diagram); the gray numbers indicate the numbers of arcs. Probe sets that are common to all three lists are represented by green arcs (15 probe sets representing eight genes). Probe set lists were obtained using a general linear model with p<.05, with the requirement that the log_2_ signal was required to be >5 for at least one of the sample groups and the absolute fold change was required to be greater than 1.5. Only annotated probe sets are depicted. For this figure, the larger fold change was used for the two time points after baseline.

The number of significant annotated genes for either 2 or 10 week relative to baseline whose corresponding probe sets met our statistical criteria (see Methods) for RA, PsA and Ps in CD14^+^ and CD14^−^ cells are shown in Table 2. These lists of significant genes that changed in response to IFX had limited overlap, and thus were fairly distinct. Figure 2 illustrates this finding for CD14^+^ cells as a hive plot which reveals more information about the overlap than a Venn diagram. Each shaded bar emanating from the center represents an ordered arrangement of probe sets color-coded according to fold-change relative to baseline. Arcs connecting the bars indicate probe sets present in two lists (green if in all three). The small number of arcs as compared to the lengths of the gene lists (indicated within each bar) reflects the low overlap between pairs of lists. Furthermore, when there is overlap, it tends to be concentrated for genes with large fold changes.

**Figure 3:**
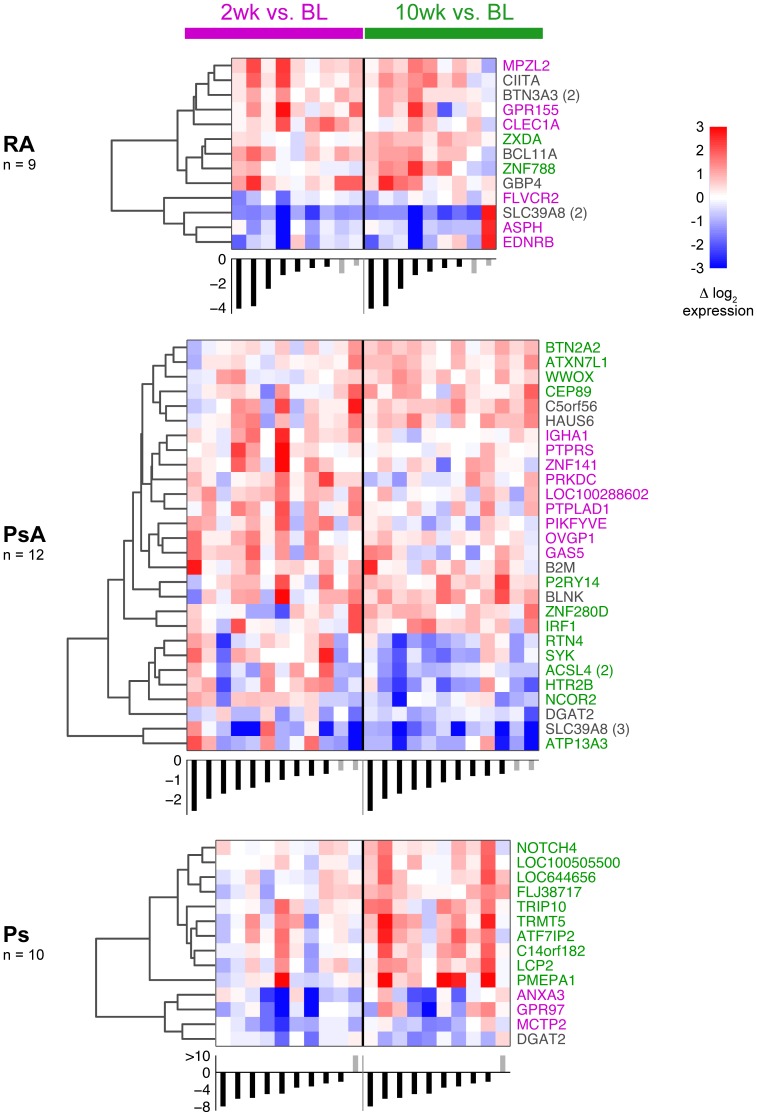
Heat map of IFX treatment effect for CD14^+^ cells in different diseases. In each heat map, columns correspond to patients and rows to probe sets. Separate heat maps are shown for three diseases and two cell types as indicated. Color is the log_2_ difference in expression at 2 or 10 weeks relative to baseline as specified by the blue and gold bars, respectively (patients are in same order for each set of columns for each disease). Probe set clustering was accomplished using Euclidean distance and complete linkage. Only genes for which p<.005 for one of the time points are shown. BL = baseline. Font color for gene names indicate whether the probe set was significant at p<.05 at 2 weeks (purple), 10 weeks (green) or both (gray). For all heat maps, the columns (patients) are sorted first by responders/non-responders, and then by change in disease activity at 10 weeks relative to baseline (DAS28 for RA and PsA, PASI for Ps), which is shown beneath each heat map (responders are black bars, non-responders are gray bars).

Figure 3 is a heat map showing the response to IFX at both time points in all three diseases for a more stringent subset of the significant genes, although note that outliers are present in each group. For all diseases, most genes shown are different at either 2 or 10 weeks, with a minority significant at both time points. Additionally, for all diseases, most of the significant genes are up-regulated in response to IFX (see also Figure 2). A similar display of data is shown for CD14^−^ cells in Figure S3. For these cells, PsA had substantially more changed genes, mostly at 10 weeks and down-regulated.

### IFX response in biopsy samples

**Figure 4:**
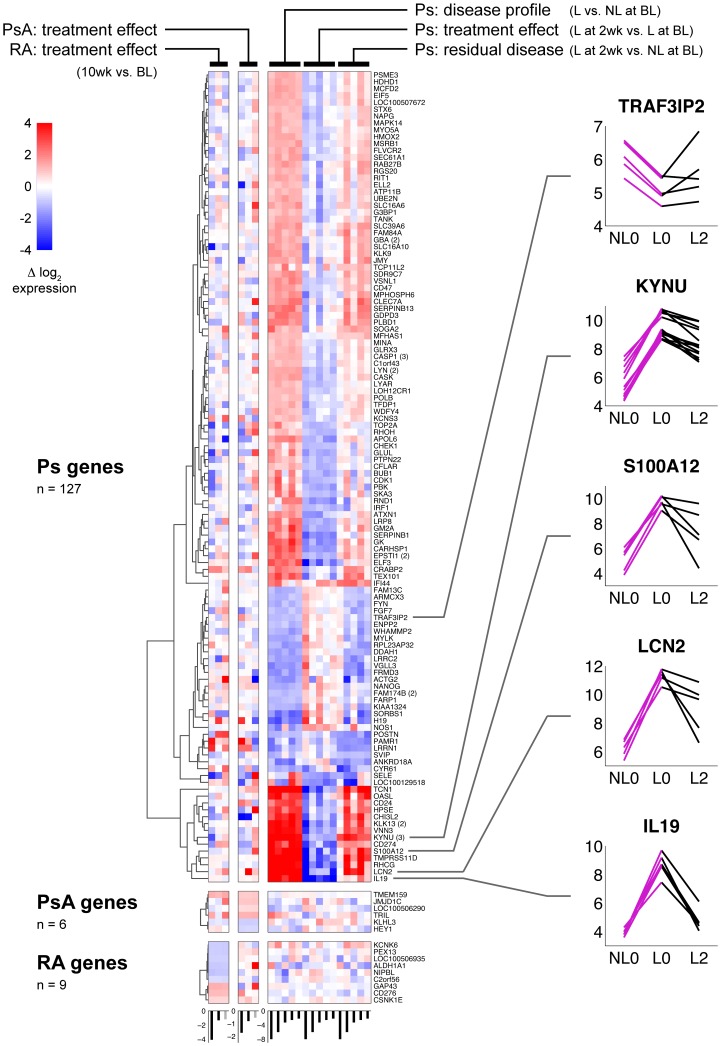
Heat map of IFX treatment effect in tissues. Paired t-tests were used to identify significant probe sets in different tissues. For Ps, comparisons, probe sets were selected where p<0.0005 and there was at least a 2-fold change in expression. For RA and PsA, paired t-tests were used and probe sets were selected where p<0.001 with at least a 1.5-fold change. Ps, PsA and RA probe set lists comprise the sets of rows, and RA, PsA and Ps samples comprise sets of columns. Color is the log_2_ difference in expression. Probe set clustering (Euclidean distance, complete linkage) was based on the corresponding sample type. BL: baseline, L: lesional, NL: non-lesional skin. Five genes are highlighted: each line is a patient; violet, black segments are cross-sectional, longitudinal comparisons, respectively. Within each of the five groups of columns corresponding to the five comparisons at the top, patients (individual heat map columns) are sorted by change in disease activity. See legend for Figure 3 for details.

Gene expression data from biopsy samples were available in a limited set of patients and is shown in Figure 4. Three gene lists are depicted: Ps genes (n = 127) which are the union of significant genes from the three possible pairwise comparisons, and PsA genes (n = 6) and RA genes (n = 9) derived from the respective knee biopsy samples. Each columnar block in the heat map represents the five possible paired comparisons (one each for RA and PsA, three for Ps, as noted in Figure 4). Large significant differences were observed in genes from Ps biopsy samples as indicated by the darker shades of red/blue. Responses are shown as insets for five selected genes significant for Ps that are relevant because they have established associations with Ps based on genome wide studies or transcriptome analyses [29]–[32]. Responses of these genes for all samples are shown in Figure S4. Note that Ps genes did not appear to have consistent expression patterns for the individuals in RA or PsA. Similarly, the PsA and RA genes, which appeared to have much weaker effects in the PsA and RA comparisons, respectively, showed no consistent pattern in Ps; the end-organ responses to the diseases were distinct.

**Figure 5:**
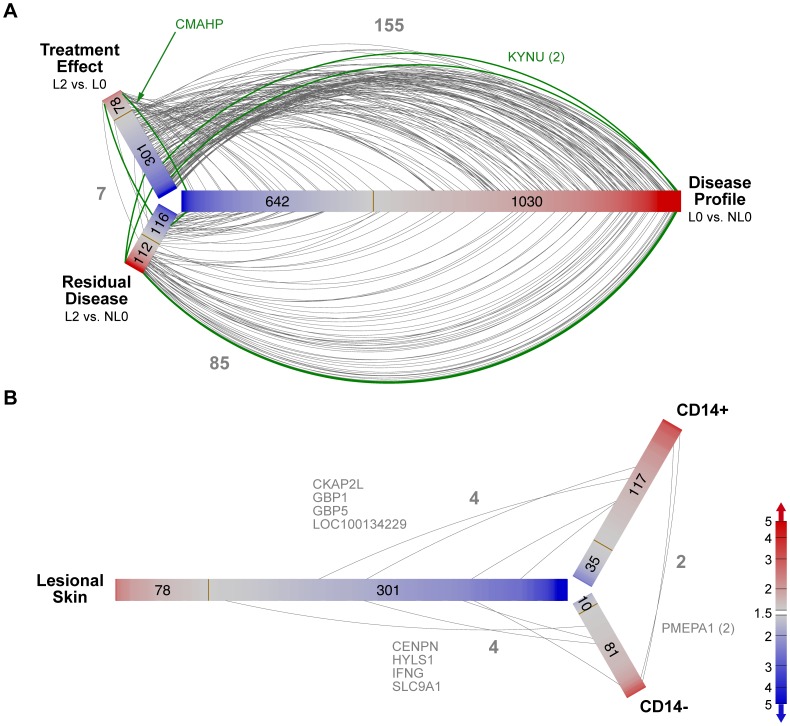
Venn/hive plots showing genes common in multiple lists for Ps samples. A. Comparisons of Ps gene lists based on skin biopsy samples. B. Comparisons of Ps Infliximab response gene lists from CD14^+^, CD14^−^ and biopsy samples. In each plot, the three shaded bars emanating from the center each represent a list of significant probe sets where their members are distributed along the bar sorted by fold-change (indicated by color). The numbers of genes up- and down-regulated are shown on the red and blue portions of the bars, respectively. Gray arcs that connect pairs of shaded bars represent genes that are common between two comparisons (in the same fashion of the intersect of two circles in a Venn diagram). Probe sets that are common to all three lists are represented by green arcs (3 in A, 0 in B). For skin biopsy comparisons, probe set lists were derived from five patients using paired t-tests based on five subjects with p<.01. For primary cell comparisons, a general linear model was used with p<.05 for ten patients. In both cases, the log_2_ signal was required to be >5 for at least one of the sample groups and the absolute fold change was required to be greater than 1.5. The false discovery rates for the biopsy samples in A, starting at the top and going clockwise, are estimated to be up to approximately 27%, 5% and 35%.

The overlap of Ps genes for biopsy comparisons are shown as a hive plot in Figure 5A at a more permissive statistical threshold than in Figure 4. Unlike the CD14^+^ or CD14^−^ comparisons which did not show strong statistical changes when accounting for multiple comparisons, the Ps biopsy comparisons at p<.01 had estimated false discovery rates of up to approximately 5% (disease effect, 3 O’clock), 27% (treatment effect, 10 O’clock) and 35% (residual disease effect, 7 O’clock). Note that all probe sets (n = 155) that are in both the disease profile list (n = 1672) and the treatment effect list (n = 379) have reciprocal directions of change; those that are up-regulated in lesional tissue relative to non-lesional tissue before treatment are down-regulated in lesional tissue following treatment, and vice-versa. When comparing the disease profile (n = 1672) with the residual disease effect (n = 228) which are genes that are different between treated, lesional tissue and pre-treated, non-lesional tissue (7 O’clock), overlapping probe sets (n = 85) always are changed in the same direction. These genes do not appear to return to a non-lesion pattern of expression. Figure 5B shows the overlap of significant probe sets for both circulating cells (CD14^+^ and CD14^−^) as well as the end organ (lesional skin) for Ps in response to IFX. The transcriptomic response to IFX appears to be unique to each compartment. These data demonstrate that peripheral blood profiles do not provide a valid assessment of gene response changes in psoriatic skin.

### Distinct enriched functions in cells and tissue in response to IFX

**Figure 6:**
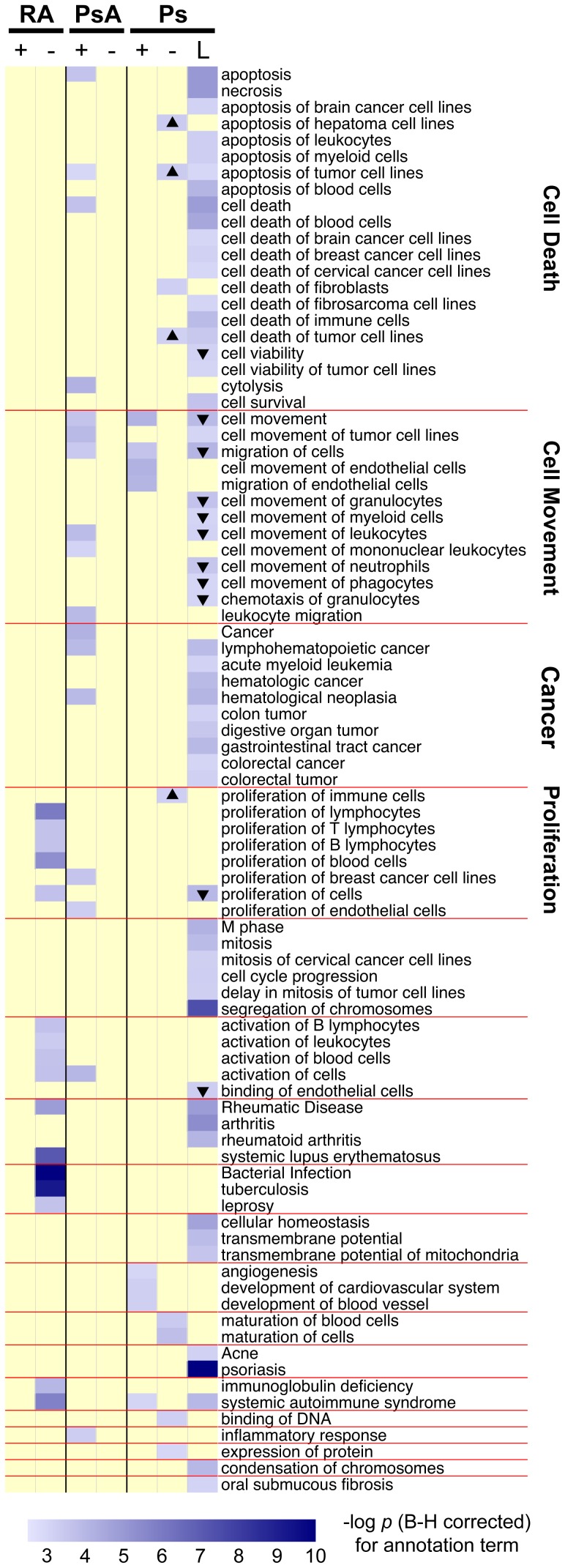
Summary of enriched functions for IFX responses for different tissues and diseases. For each of seven pre/post IFX gene lists, results from Ingenuity Pathway Analysis functional enrichment were retained if the Benjamini-Hochberg-corrected p value was <0.001 and if there were more than four probe sets associated with the function. The unions of these functions are listed alongside of the heat map. Color maps to –log_10_ of the corrected p-value, with blue being very significant. Functions (rows) were clustered based on their associated broader, category labels using the kappa statistic in order to group similar functions together to aid in visualization. Up and down triangles indicate functions that were increased or decreased, respectively, based on activation z-score.

Enriched functions derived from IPA for the IFX transcriptomic response in the three diseases are shown in Figure 6. In Ps lesional skin, the most enriched functions were Ps, segregation of chromosomes, arthritis and necrosis with many other functions related to cell death and cell movement. For Ps, in CD14^−^ cells, enriched functions are related to cell maturation and cell death, whereas in CD14^+^ cells, functions related to cell migration and blood vessel development appear to be enriched. For genes involved in the IFX response in PsA, only CD14^+^ cells showed enriched functions, largely related to inflammatory response, migration and apoptosis. In contrast, only RA CD14^−^ cells showed enriched functions following IFX treatment for immune response, activation, proliferation and autoimmune disease. Taken together, these diverse patterns of enriched function parallel the divergent transcriptomic response in the three diseases.

### Key upstream regulator involvement following IFX therapy in psoriatic skin and peripheral blood

The predicted involvement of IFX transcriptional regulators based on 379 probe sets that were significantly changed in psoriatic skin following treatment is shown in Table 4 (and with the target genes in Table S1). This analysis identifies a potential upstream regulator if the list of genes is enriched for targets of the regulator in question, and if the pattern of up- and down-regulation is consistent with an activation state [26]. Eight transcription factors whose targets overlapped with genes represented by the input probe set list with p<.0001 (based on IPA upstream regulator analysis) are listed, and those whose targets changed in directions consistent with an activated or inhibited state are shaded orange or pink, respectively. Targets that correspond to probe sets in the input list are shown as well, along with their direction of change in response to IFX. With the exception of STAT3, none of the predicted regulators themselves were changed, although, of the target genes represented in the significant gene list, the vast majority were down-regulated. Note that FOX01 [33], STAT3 [34], RELA [35] and JUN [36], transcriptional regulators important in cell proliferation and differentiation, show inhibition in contrast to KDM5B, a histone demethylase that blocks terminal differentiation [37] and TP53, an inducer of cell death [38] which show activation.

**Table 4:** Upstream regulator analysis using Ingenuity Pathway Analysis.

Regulator	Changed	z-Score	Predicted State	Bias	−log_10_ p-Value
TP53	−	3.25	activated	−	11.89
FOXO1	−	−3.29	inhibited	yes	9.7
JUN	−	−1.28	−	yes	7.07
FOXM1	−	−2.56	inhibited	yes	7.01
RELA	−	−2.62	inhibited	yes	5.34
TP63	−	−1.59	−	yes	5.2
KDM5B	−	2.99	activated	yes	4.33
STAT3	down	−0.48	−	−	4.19

Upstream regulators predicted based on genes that were significantly different for lesional skin before and after treatment with IFX (n = 379). Subset of “significant” transcription factors are shown that had an overlap p-value <.0001 (negative log of 4) and a reported activation z-score, indicating that many of the targets were enriched in the gene list. Except for STAT3 which was down-regulated, none of these were in the gene list. The further the activation z-score is from zero, the more likely that the direction of change of the target genes are consistent with the regulator being in either an “activated” or “inhibited” state. Bias terms over 25 are shown, indicating that the regulation of the targets in the data set as well as all of those for the regulator are skewed towards a particular direction [26]. Six additional upstream regulators, E2F4, E2F1, PBRM1, NFYB, MYBL2 and NFkB, had low overlap p-values, but there wasn’t sufficient data about known relationships with targets to predict an activation state. Although no upstream regulator was predicted to be activated or inhibited for the IFX-response in CD14^+^ or CD14^−^ cells, some still had low overlap p-values.

Evidence for upstream regulator involvement was weaker in peripheral blood, and in no case was there predicted activation or inhibition. For the following results, a more permissive threshold of p<.001 was used. In RA, CD14^+^ cells showed enrichment for interferon regulatory factor IRF4 and SMARCA4 [39] a chromatin remodeling protein while CD14^−^ cells were enriched for CEBPE [40], which regulates myeloid differentiation and musculin (MSC) [41], a transcription factor expressed in skeletal muscle. In PsA, CD14^+^ cells were enriched for CEBPA, a transcription factor critical for granulocyte differentiation [42], while CD14^−^ cells showed no suggested involvement for a particular transcription factor. In Ps, CD14^+^ cells, AR met the significance threshold while in CD14^−^ cells, RORC, a transcription factor in Th17 cells [43], NR3C1, a glucocorticoid gene with polymorphisms linked to obesity, hypertension, elevated lipid levels and increased cardiovascular risk [44], and PYCARD-an adaptor protein for NLRP3 the inflammasome forming protein [45] had the lowest p values. Note that none of these regulators found in Ps blood were in common with those listed in Table 4.

## Discussion

The immune mediated inflammatory diseases RA, PsA and Ps have divergent phenotypes but share several pathologic features that include overproduction of TNF and other cytokines, along with cellular proliferation and tissue remodeling. They also have overlapping metabolic and cardiovascular comorbidities and demonstrate, on average, good to excellent responses to anti-TNF agents. We hypothesized that the genomic response to anti-TNF agents in these three diseases would be distinct and would vary in the different cell types and target tissues. To our knowledge, this is the first study of differential gene expression in discrete populations of monocytes and immune cells that also examined expression in target tissues following treatment with IFX. We found the effect of IFX on the number of genes expressed by CD14^+^ and CD14^−^ cells more striking in PsA compared to RA and Ps. Moreover, the response in RA was clustered around the 2 week time point whereas the most notable effect on gene expression in PsA and Ps was at 10 weeks. In Ps biopsies, a large number of genes were dramatically down-regulated by IFX at 2 weeks but the gene expression profile in skin from patients with excellent clinical responses was still significantly different from uninvolved skin. We also noted that the IFX response in both circulating CD14^+^ and CD14^−^ cells was strikingly different than the response observed in the skin or synovium of the same patients. Functional enrichment analysis revealed involvement of signaling cascades in CD14^−^ cells (B, T lymphocytes, NK cells) in RA, CD14^+^ cells (monocytes) in PsA and a mixture of these different cell lineages in Ps. Moreover, little overlap was noted in the 3 diseases regarding involvement of specific functional pathways in the IFX response. These data suggest that although TNF is of central importance in all three diseases based on the excellent clinical responses, the pathways modulated by TNF differ suggesting distinct disease mechanisms. Finally, a number of key transcriptional regulators were either activated or suppressed after treatment but little overlap was observed between the disorders in terms of specific regulators involved.

Overall, analysis of peripheral blood resulted in significant gene lists that were not maintained following corrections for multiple comparisons. As a result, determination of specific transcription factor involvement in blood was also limited. This could be due in part either to more subtle transcriptional changes in the blood, or to the heterogeneous nature of the CD14^+^ and, especially, CD14^−^ subsets. Ps skin biopsy samples gave strong transcriptional responses, whereas knee biopsy samples in PsA and RA resulted in less clear-cut signatures, most likely due to the sample size. The data support the concept that PsA is more dependent on innate immune responses mediated largely by monocytes (CD14^+^) in contrast to RA which is driven largely by an acquired immune signature (CD14^−^) expressed by T cells, T lymphocytes and NK cells while Ps reveals involvement of both immune mechanisms (CD14^+^ and CD14^−^).

The high number of genes in CD14^+^ and CD14^−^ cells with significant changes in expression following IFX treatment in PsA compared to RA and Ps samples was unexpected. Also, the largest effect on gene expression was noted at week 10 in PsA and Ps compared to 2 weeks for most of the genes in the RA samples. This may explain why skin responses often lag behind the joint response to anti-TNF agents in PsA. In addition, changes in the number of genes altered by IFX showed greater similarity in RA and PsA compared to Ps. One potential explanation is that the majority of RA and PsA patients were on methotrexate prior to IFX therapy whereas none of the Ps patients were on this medication at baseline. Two other studies examined cross-sectional gene expression in 29 PsA patients and controls. Batliwalla et al. [46] found that 257 genes were expressed at low and 56 at elevated levels compared to controls. They found low levels of several genes that suppress innate and acquired immune responses and upregulation of 3 proinflammatory genes (S100A, S100A12) and thioredoxin. The gene signature for PsA was different than RA. In another study, Stoeckman et al. found 310 differentially expressed genes in 16 PsA patients compared to controls, most of which were upregulated [47]. The signature differed from those found in RA and SLE but interestingly showed little overlap with Batliwalla et al. [46]. We cannot directly compare our findings with these 2 studies, since we examined the change in gene expression after IFX, but we did not see overlap with our gene lists and these two data sets. The reason for the higher number of differential gene expression in PsA compared to RA and Ps after IFX may relate to the fact that inflammatory cells that transit to both the skin and joint may be triggered by an array of signals that activate numerous gene networks. Further study is required to address this finding, however.

Transcriptome analysis of Ps lesional skin showed marked differential gene expression compared to non-lesional skin at baseline. Our findings strongly correlate with the results of a Ps transcriptome meta-analysis recently published by Tian et al [28] [39](see Figure S5). In this meta-analysis of 5 microarray data sets, 1832 differentially expressed genes in Ps lesional compared to non-lesional skin were identified (1084 up and 748 down) similar to our results (1672 differentially expressed probe sets: 1030 up and 642 down). Of the top 50 differentially regulated probe sets (25 up and 25 down) reported by this group, we found 48 of the 50 probe sets in our dataset had fold changes in the same direction. For those that were up (L0 vs. NL0), 21 of the 25 probe sets were significant at p<.01. For those that were down, 6 of the 23 were significant at the same level. Most strikingly, we found that all of these 25 genes up-regulated at baseline were down-regulated 2 weeks after IFX treatment (p<.05 for 12 of those; for all the 1030 probe sets up-regulated in L0 relative to NL0 in our data set, 131 were down-regulated at L2 as depicted in Figure 5A). Despite the suppression of these genes following treatment, lesional skin showed differential up-regulation (112 genes) and down-regulation (116 genes) in treated L skin compared to NL skin. This residual disease genomic profile or ‘molecular scar’ was also noted in psoriatic lesions after treatment with etanercept [48]. It represents continued activation of cytokines and chemokines in skin where Ps has phenotypically resolved. Of their 20 selected, up-regulated disease genes that didn’t show “improvement” following etanercept, five overlapped with our L2 vs. NL0 up-regulated list for at least one probe set (RAB31, WNT5A, SYK, IFI27 and ISG20, with IFI27 and WNT5A having the most residual response). Of their 9 selected, down-regulated disease genes, only AQP9 overlapped with our L2 vs. NL0 down-regulated list. The other important finding is that only 8 probe sets differentially expressed after IFX treatment, were shared between the lesional skin and any of the blood cells, and with fairly small fold-changes. Thus, our findings confirm those presented in the meta-analysis and demonstrate a dramatic suppression of key differentially expressed genes as early as two weeks following IFX therapy, however, residual inflammatory pathways remain active, despite apparent skin response.

Several transcription factors were predicted to be involved following treatment in the skin and blood cells of the 3 patient groups included in the study based on the significant overlap of associated gene lists and known upstream regulator [26]. In Ps plaques, transcription factor genes that promote cell differentiation and proliferation including STAT3, RELA and JUN were predicted to be inhibited. In contrast KDM5B a histone demethylase and TP53 a suppressor oncogene was predicted to be activated after treatment. The effect of treatment on the blood cells in RA was more limited and upstream-regulator involvement was noted for SMARCA4 and ILF4 in CD14^+^ cells and CEPBE and MSC in CD14^−^ cells. In PsA, the only enrichment was noted for CEBPA in CD14^+^ cells. These findings indicate that the effect of IFX is far more robust at the tissue level and that changes in the blood transcriptome do not correlate well with changes in the tissue.

The enriched gene functions for IFX responses in the 3 disorders were of particular interest and likely reflect contrasting disease mechanisms and/or differing treatment responses to IFX. In reviewing the pathway differences between RA and PsA, it is apparent the response to IFX is most notable only in RA CD14^−^ cells. Pathways affected are those related to proliferation of lymphocytes, T cells, B cells, bacterial infection, tuberculosis, SLE and systemic autoimmune syndrome among others. In the case of PsA, pathways that changed were limited to the CD14^+^cells and included proliferation of cells and endothelial cells, apoptotic pathways, inflammatory response, cell movement and migration. In contrast, significant treatment changes were observed in pathways associated with both CD14^+^ and CD14^−^ cells in psoriatic peripheral blood. CD14^+^ associated pathways noted to be affected were those associated with angiogenesis, blood vessel development, autoimmunity, cell movement and migration. CD14^+^ pathways significantly impacted by IFX in psoriasis were proliferation of immune cells, apoptosis and cell death, and maturation. The longitudinal data in table 2 demonstrate that IFX therapy at 10 weeks was associated with a more pronounced effect on the number of genes associated with CD14^−^ compared to CD14^+^ cells in PsA and contrasts with the predominance of genes expressed by CD14^+^ cells outlined in the pathway analysis outlined above. It is important to note that the numbers of differentially expressed genes do not always correspond to enriched pathways derived from gene lists which are dependent on specific genes that comprise the pathway. The results from the pathway analysis support a disease paradigm put forth by McGonagle et al. in which RA pathogenesis is mediated largely by acquired immune mechanisms, PsA primarily by the innate immune response, and Ps by a combination of acquired and innate pathways, but further studies with larger sample sizes are required to confirm this model [49].

Our study is the first examination of differential gene expression in cell populations and target tissues from different immune mediated inflammatory disorders but several limitations must be considered. First, the low number of synovial samples in diseases with heterogeneous tissue responses does not provide a comprehensive assessment of the joint response although the relationship between differential gene regulation in the joint and skin of an individual patient is informative. Second, we do not have confirmatory immunohistochemistry data on tissue expression of differentially expressed genes determined by microarray. Third, the lack of a sufficient number of non-responders to IFX (for RA there were two overall, one in the biopsy patient set) did not allow us to identify genes that may be responsible for persistent inflammation following treatment. Fourth, microarray studies are directed towards analysis of defined genes and increasing evidence points to the importance of critical regulatory elements outside the protein-coding regions which will not be captured with this approach [39].

In conclusion, these results indicate that anti-TNF treatment modulates different disease pathways in specific immune-mediated inflammatory disorders. Thus, clinical and tissue responses can be achieved with TNF inhibition by divergent mechanisms dependent on the underlying disease. Moreover, careful analysis of differential gene expression in involved tissues and possibly blood cells following treatment with biologic therapies may provide insights into disease pathogenesis and unveil new disease targets.

## Supporting Information

Figure S1 **Heat map of cross-sectional expression data for CD14^+^ cells.** In each heat map, columns correspond to patient samples and rows to probe sets. Color is the per-probe set z-score of log_2_ expression across all 50 baseline samples. Gene clustering was accomplished using Euclidean distance and complete linkage. Only genes where p<.0005 for one of the disease vs. healthy control (HC) comparisons are shown. Dots to the left of the probe set names indicate which comparison met this p-value threshold. Columns (patients) were sorted by baseline disease activity (DAS28 for RA and PsA, PASI for Ps) and are shown beneath the heat maps.
(TIF)

Figure S2 **Heat map of cross-sectional expression data for CD14^−^ cells.** See description for Figure S1. The high number of Hb genes noted (13 probe sets for HBA1, HBB, HBG1 and HBD in the bottom third of the heat map) in RA may be related to 1) upregulation of Hb genes (previously reported in juvenile RA [50], 2) presence of erythroid precursors 3) contamination with blood. We think the latter possibility very unlikely because all samples were handled in an identical fashion and increased expression of Hb genes was not observed in the psoriasis or PsA samples.
(TIF)

Figure S3 **Heat map of longitudinal expression data for CD14^−^ cells.** See description for Figure 3. Missing data for one 10 week PsA samples (third column, white).
(TIF)

Figure S4 **Examples of gene expression pattern across all 255 samples for genes in call-outs of** **Figure 4** **.** x-axis labels are time points. B = baseline; BN = baseline, non-lesional skin, HC = healthy control.
(TIF)

Figure S5 **Ps lesional skin vs. non-lesional skin data for 50 genes identified in Ps meta-analysis of Tian et al.** [28] **.** Log2 fold change values for the 50 genes in the meta-analysis compared to the values for the same genes in our study.
(TIF)

Table S1 **Upstream regulator analysis using Ingenuity Pathway Analysis, including target molecules.**
(PDF)

Table S2 **Validation of microarray results by RT-PCR for CD14^+^ samples in longitudinal comparisons.**
(PDF)

Table S3 **Validation of microarray results by RT-PCR for CD14^+^ samples in cross-sectional comparisons.**
(PDF)

Table S4 **Validation of microarray results by RT-PCR for biopsy samples.**
(PDF)

Table S5 **TaqMan primers used for validation.**
(PDF)
