# Supplementary figures and images for: Divergent Gene Activation in Peripheral Blood and Tissues of Patients with Rheumatoid Arthritis, Psoriatic Arthritis and Psoriasis following Infliximab Therapy
Source: PLoS One. 2014 Oct 21;9(10):e110657. doi: 10.1371/journal.pone.0110657 (PMC4204991; doi:10.1371/journal.pone.0110657)

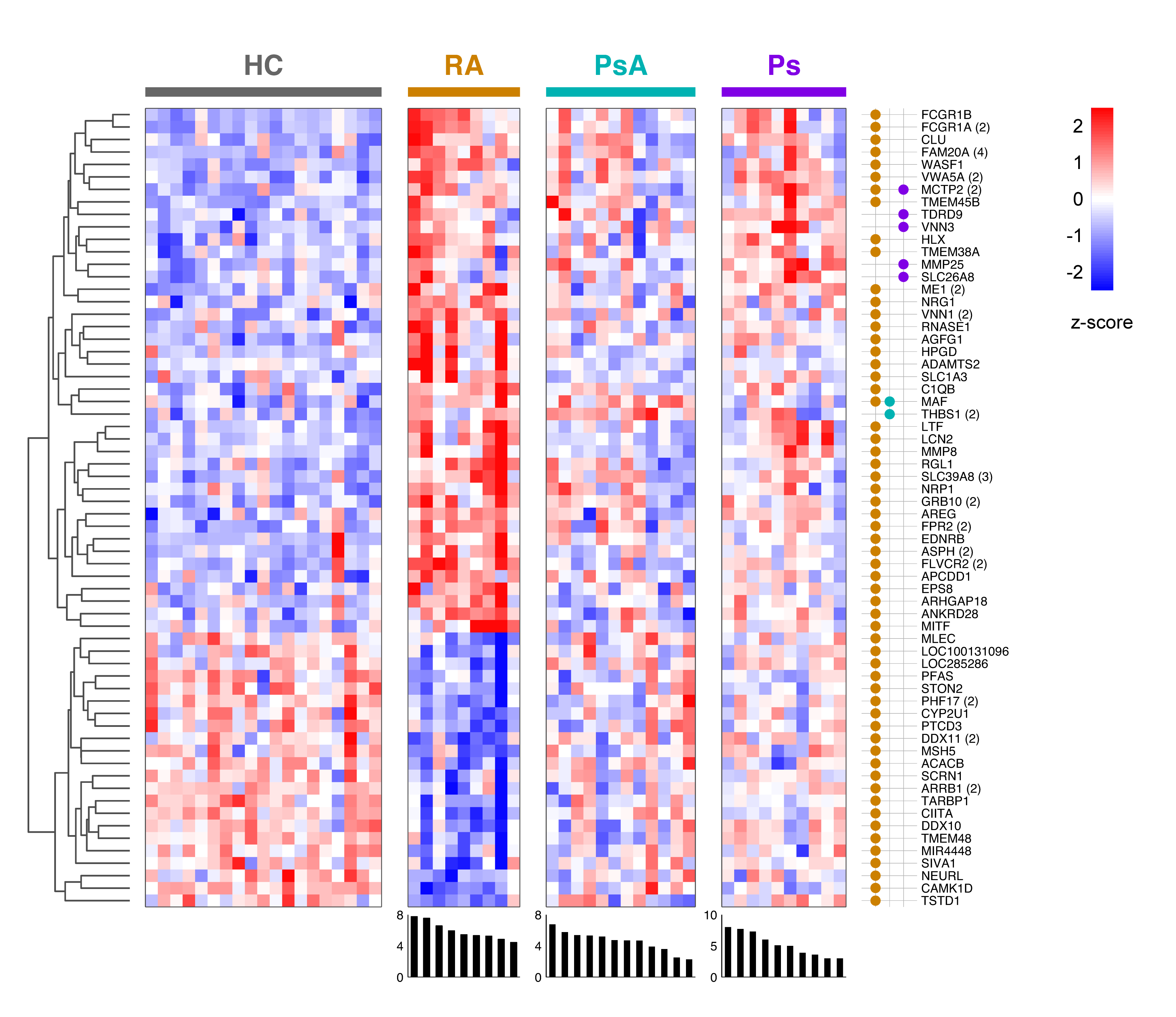

Supplement: Figure S1 — Heat map of cross-sectional expression data for CD14+ cells. In each heat map, columns correspond to patient samples and rows to probe sets. Color is the per-probe set z-score of log2 expression across all 50 baseline samples. Gene clustering was accomplished using Euclidean distance and complete linkage. Only genes where p<.0005 for one of the disease vs. healthy control (HC) comparisons are shown. Dots to the left of the probe set names indicate which comparison met this p-value threshold. Columns (patients) were sorted by baseline disease activity (DAS28 for RA and PsA, PASI for Ps) and are shown beneath the heat maps. (TIF) [file pone.0110657.s001.tif]

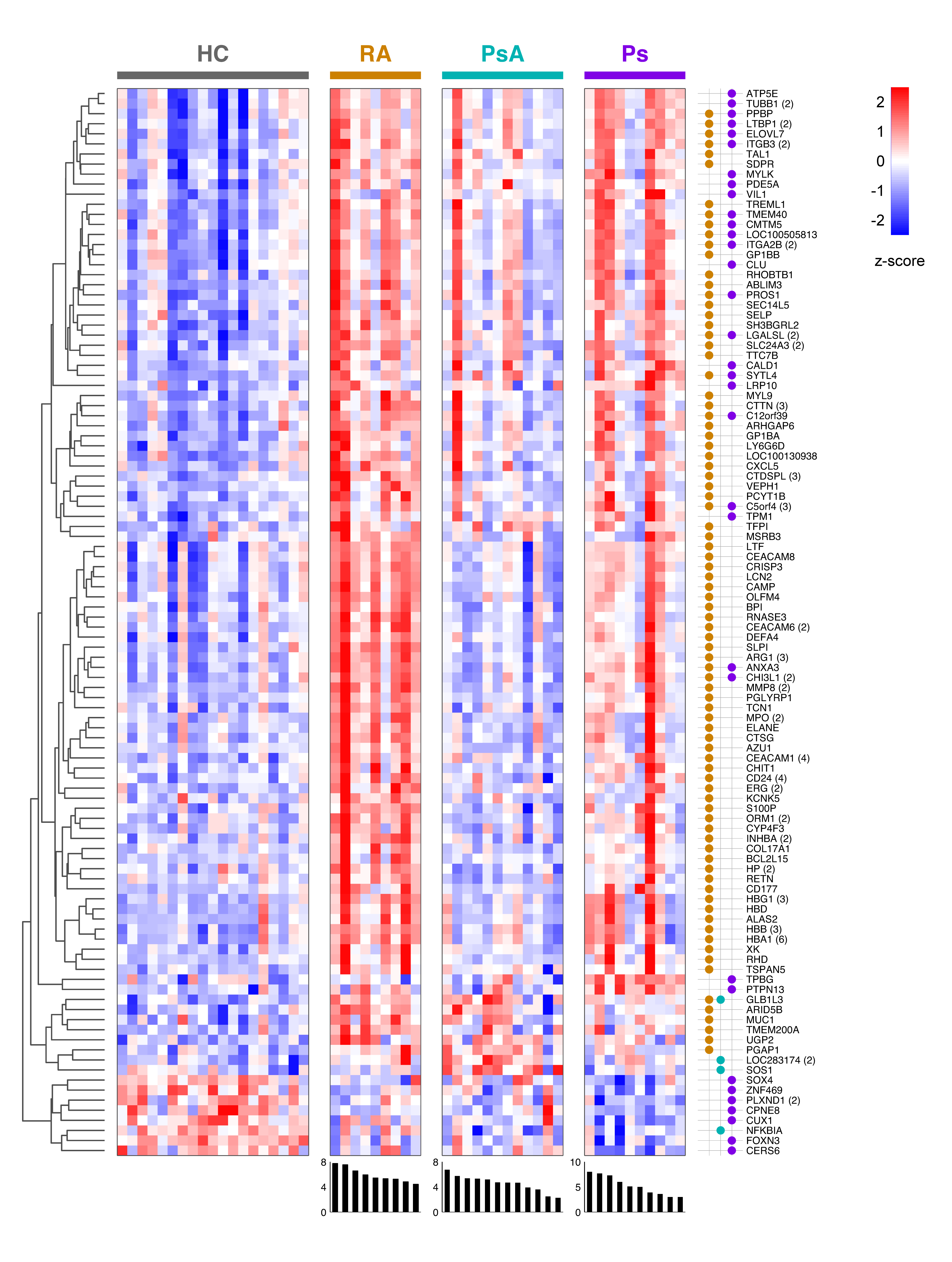

Supplement: Figure S2 — Heat map of cross-sectional expression data for CD14− cells. See description for Figure S1. The high number of Hb genes noted (13 probe sets for HBA1, HBB, HBG1 and HBD in the bottom third of the heat map) in RA may be related to 1) upregulation of Hb genes (previously reported in juvenile RA [50], 2) presence of erythroid precursors 3) contamination with blood. We think the latter possibility very unlikely because all samples were handled in an identical fashion and increased expression of Hb genes was not observed in the psoriasis or PsA samples. (TIF) [file pone.0110657.s002.tif]

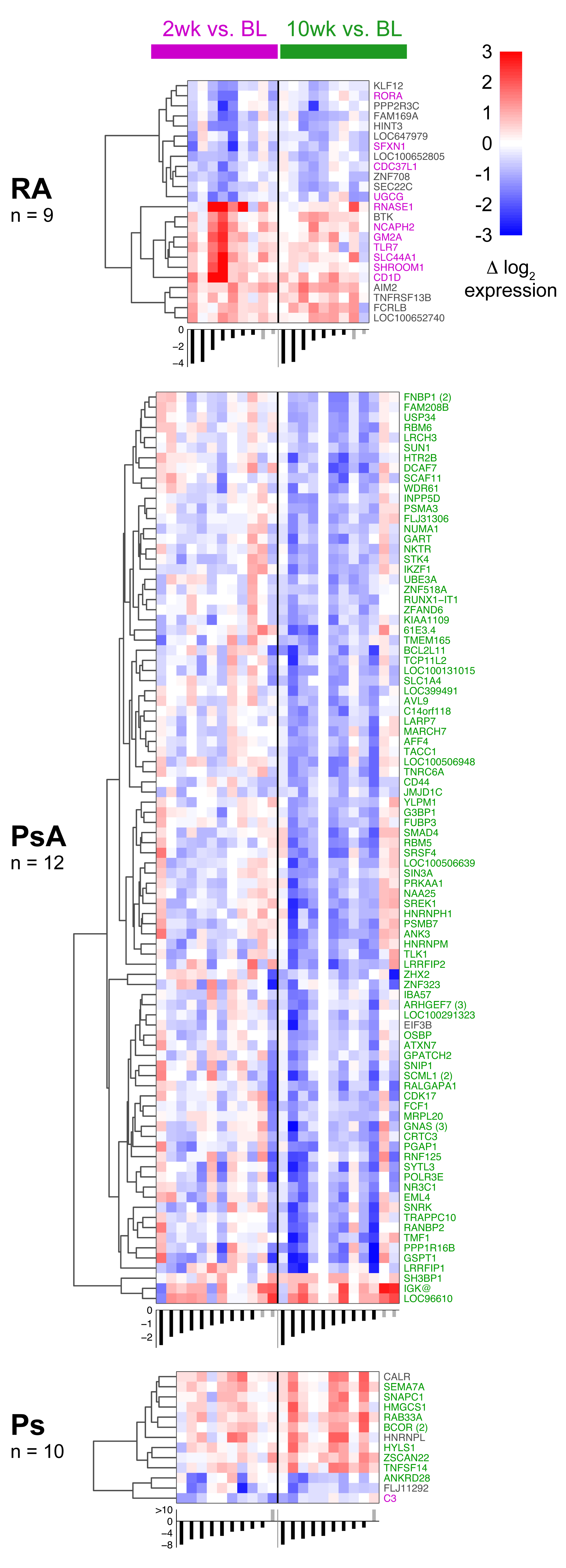

Supplement: Figure S3 — Heat map of longitudinal expression data for CD14− cells. See description for Figure 3. Missing data for one 10 week PsA samples (third column, white). (TIF) [file pone.0110657.s003.tif]

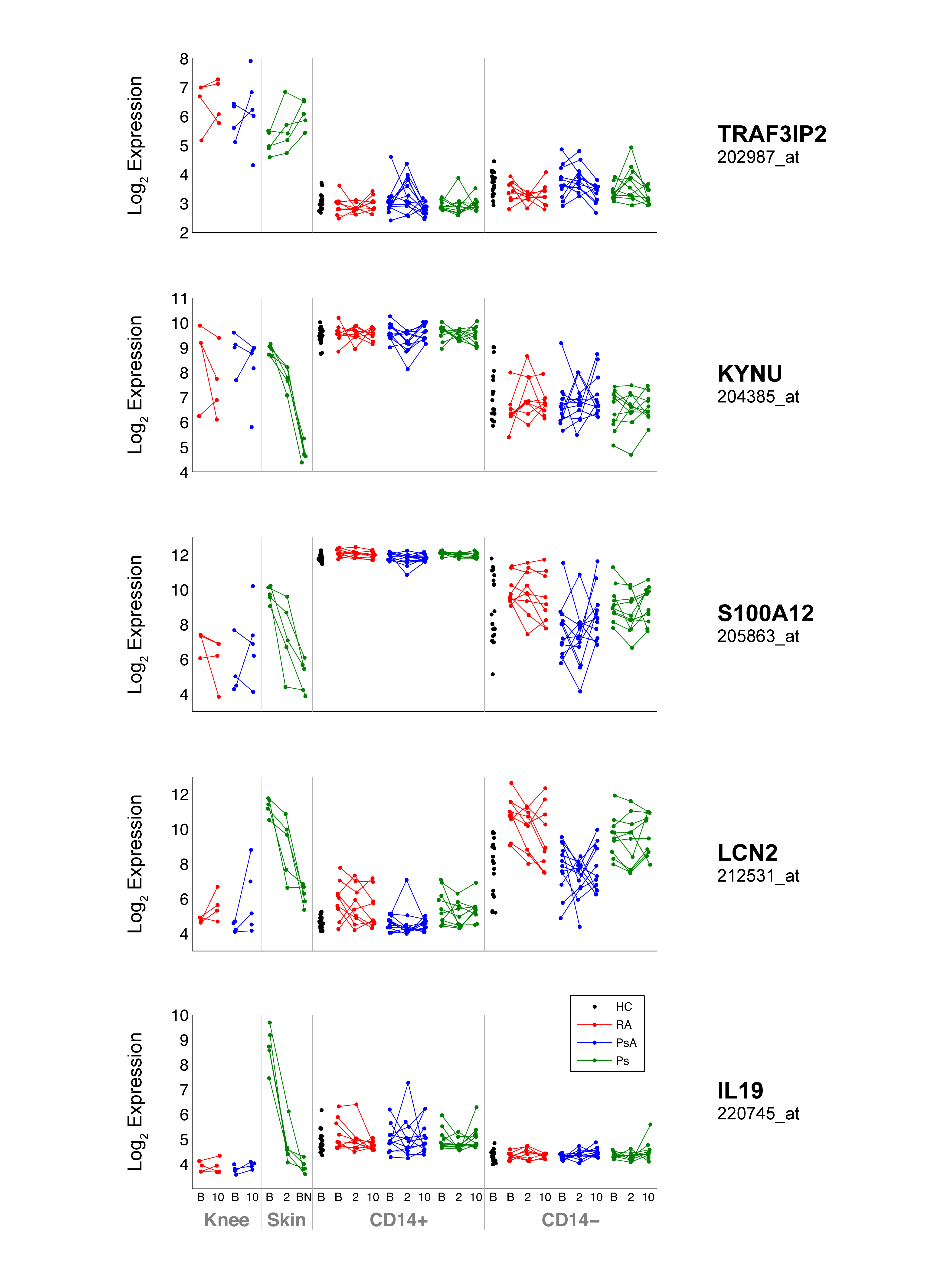

Supplement: Figure S4 — Examples of gene expression pattern across all 255 samples for genes in call-outs of Figure 4 . x-axis labels are time points. B = baseline; BN = baseline, non-lesional skin, HC = healthy control. (TIF) [file pone.0110657.s004.tif]

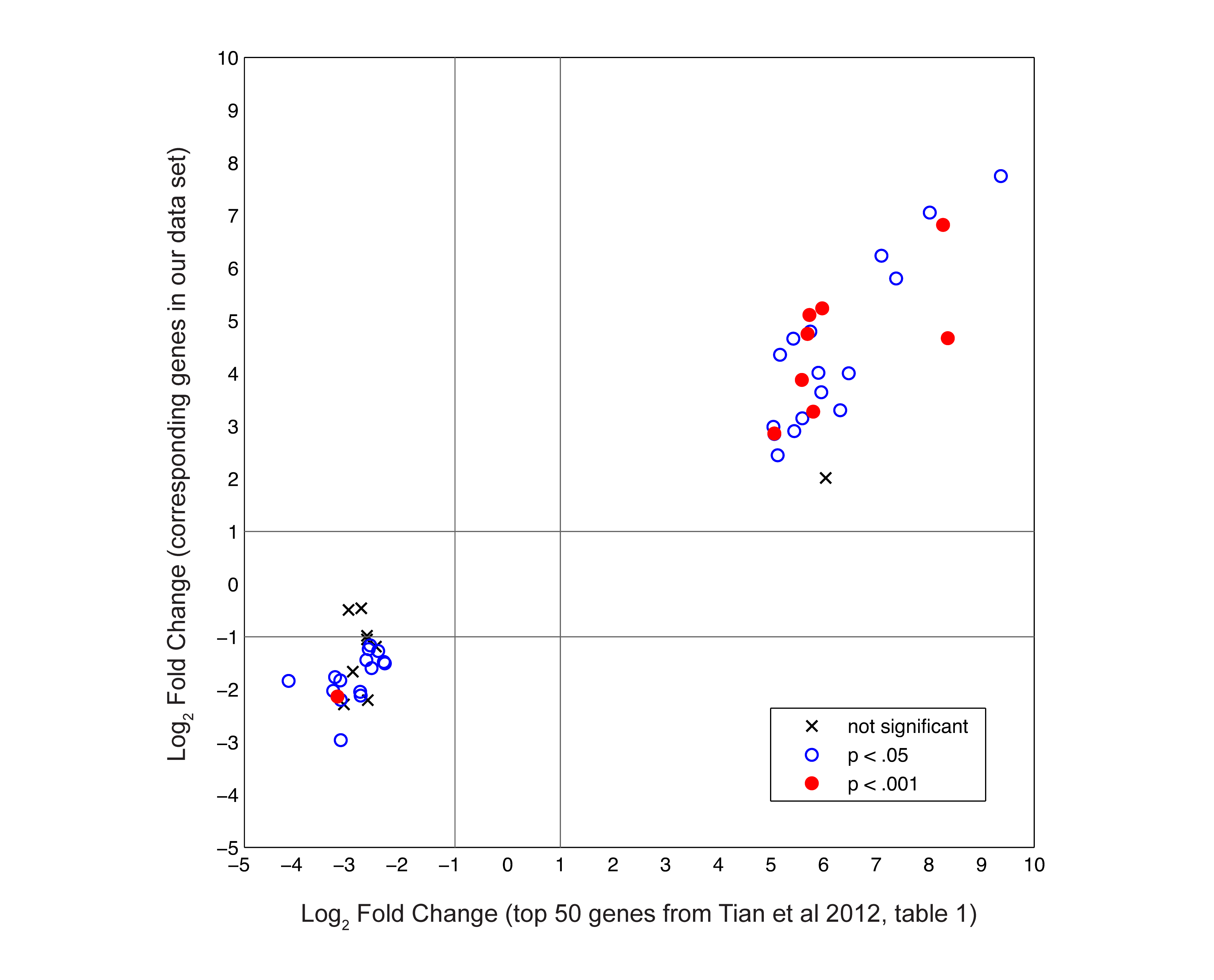

Supplement: Figure S5 — Ps lesional skin vs. non-lesional skin data for 50 genes identified in Ps meta-analysis of Tian et al. [28] . Log2 fold change values for the 50 genes in the meta-analysis compared to the values for the same genes in our study. (TIF) [file pone.0110657.s005.tif]
