# Supplementary material for: Divergent Gene Activation in Peripheral Blood and Tissues of Patients with Rheumatoid Arthritis, Psoriatic Arthritis and Psoriasis following Infliximab Therapy
Source: PLoS One. 2014 Oct 21;9(10):e110657. doi: 10.1371/journal.pone.0110657 (PMC4204991; doi:10.1371/journal.pone.0110657)
Supplement: Table S1 — Upstream regulator analysis using Ingenuity Pathway Analysis, including target molecules. (PDF) [file pone.0110657.s006.pdf]

Upstream Regulator	Activation z-Score	-log10 Overlap p-Value	Target Molecules in Ps L0 vs. L2 Gene List
**TP53**	3.25	11.89	↓ARPC1B ↓BIRC5 ↓BUB1 ↓CCNA2 ↓CCNB1 ↓CDC25A ↓CDK1 ↓CENPF ↓CEP55 ↓CHEK1 ↓DHFR ↓FEN1 ↓HMMR ↓HS3ST1 ↓KIAA0101 ↓MAD2L1 ↓NCAPG ↓NDC80 ↓NEK2 ↓PBK ↓PLAUR ↑PRKAB2 ↓RRM2 ↓SRSF3 ↓TOP2A ↓ANXA1 ↓C12orf5 ↓CASP1 ↓CXCL1 ↓GBP1 ↓SOD2 ↓CD47 ↓CDKN3
**FOXO1**	-3.29*	9.7	↓ASPM ↓BIRC5 ↓CCNB1 ↓CDK1 ↓CENPF ↓DEPDC1 ↓DLGAP5 ↓NCAPG ↓NEK2 ↓NUSAP1 ↓SPC25
**JUN**	-1.28*	7.07	↓CCL8 ↓CCL2 ↓ICAM1 ↓IFNG ↓SOD2 ↑ALOX12 ↓CD274 ↓CDK1 ↓FTH1 ↓IL7R ↑VAV3
**FOXM1**	-2.56*	7.01	↓BIRC5 ↓CCNA2 ↓CCNB1 ↓CDK1 ↓CDKN3 ↓CENPF ↓GTSE1 ↓NEK2
**RELA**	-2.62*	5.34	↓CXCL1 ↓ICAM1 ↓IRF1 ↓LYN ↓NAMPT ↓SAA2 ↓SOD2 ↓CXCL9 ↓ELF3 ↓IL1RN ↓IL20
**TP63**	-1.59*	5.2	↑F3 ↓TNC ↓CCNA2 ↓COL4A1 ↓HAS3 ↓LYN ↓MAD2L1 ↓MCM10 ↓PNPT1 ↓RAD51 ↓CDK1
**KDM5B**	2.99*	4.33	↓BUB3 ↓CCNB1 ↓CDK1 ↓DLGAP5 ↓HMMR ↓NCAPH ↓NDC80 ↓PBK ↓TOP2A
**↓STAT3**	-0.48	4.19	↓CXCL9 ↓IRF1 ↑MAP2K5 ↓CCL2 ↓NAMPT ↓PHB ↓STAT3 ↓IL1RN

predicted regulator activation state:

based on all targets: (orange box) activated; (pink box) inhibited

based on particular target: target1 activated; target2 inhibited

Supplemental Table S1. Upstream regulator analysis using Ingenuity Pathway Analysis, including target molecules. Upstream regulators predicted based on genes that were significantly different for lesional skin before and after treatment with IFX (n = 379). Subset of "significant" transcription factors are shown that had an overlap p-value < .0001 (negative log of 4) and a reported activation z-score, indicating that many of the targets were enriched in the gene list. Up- and down-regulation of particular target genes are specified by red and blue arrows, respectively. The further the activation z-score is from zero, the more likely that the direction of change of the target genes are consistent with the regulator being in either an "activated" or "inhibited" state. The directional relationship between regulator and target is not specified for genes in black font. Asterisks indicate a bias term over .25, indicating that the regulation of the targets in the data set as well as all of those for the regulator are skewed towards a particular direction ["Ingenuity Upstream Regulator Analysis in IPA" white paper, Ingenuity Systems]. Six additional upstream regulators, E2F4, E2F1, PBRM1, NFYB, MYBL2 and NFkB, had low overlap p-values, but there wasn't sufficient data about known relationships with targets to predict an activation state. Although no upstream regulator was predicted to be activated or inhibited for the IFX-response in $CD14^+$ or $CD14^-$ cells, some still had low overlap p-values.
