# Supplementary material for: Divergent Gene Activation in Peripheral Blood and Tissues of Patients with Rheumatoid Arthritis, Psoriatic Arthritis and Psoriasis following Infliximab Therapy
Source: PLoS One. 2014 Oct 21;9(10):e110657. doi: 10.1371/journal.pone.0110657 (PMC4204991; doi:10.1371/journal.pone.0110657)
Supplement: Table S2 — Validation of microarray results by RT-PCR for CD14+ samples in longitudinal comparisons. (PDF) [file pone.0110657.s007.pdf]

CD14+ LONGITUDINAL COMPARISON VALIDATION

Disease	Gene	TaqMan				Microarray				Same Direction at 2w	Same Direction at 10w	TaqMan Validates at One Time Point
		2 week FC	2 week raw p	10 week FC	10 week raw p	2 week FC	2 week raw p	10 week FC	10 week raw p			
RA	CIITA	1.82	0.00089	1.88	0.00391	1.67	0.00480	1.67	0.00500	1	1	1
	CKAP2	1.87	0.00460	2.09	0.00092	1.66	0.10060	2.32	0.00720	1	1	1
	GBP4	3.04	0.00806	2.24	0.04079	2.02	0.00190	1.73	0.01480	1	1	1
	IGLC1	2.66	0.04241	1.70	0.38857	1.73	0.15970	2.78	0.00940	1	1	1
	IGKC	2.33	0.43120	5.10	0.19321	2.02	0.07870	2.63	0.01660	1	1	0
	SLC39A8	-1.97	0.00051	-2.08	0.07111	-3.35	0.00200	-2.59	0.01390	1	1	1
PsA	BLNK	1.13	0.78872	1.74	0.04095	1.57	0.02730	1.88	0.00230	1	1	1
	CKAP2	1.05	0.87184	1.79	0.02245	1.72	0.04230	1.42	0.19250	1	1	1
	GBP4	2.68	0.31815	4.14	0.07028	1.32	0.15110	1.71	0.00580	1	1	0
	IGLC1	2.00	0.33158	2.69	0.04501	2.4	0.01680	2.09	0.04230	1	1	1
	IGKC	-1.07	0.94539	3.50	0.38979	2.14	0.02020	1.92	0.04560	0	1	0
	SLC39A8	-2.18	0.05417	-3.42	0.00010	-2.18	0.03510	-3.61	0.00070	1	1	1
Ps	CKA2PL	1.28	0.53195	2.40	0.02126	1.15	0.54200	1.86	0.00660	1	1	1
	CKAP2	1.47	0.00284	1.22	0.34195	2.04	0.01570	1.14	0.65200	1	1	1
	GBP1	1.35	0.32588	1.66	0.12973	1.29	0.29890	1.65	0.04620	1	1	0
	GBP4	1.31	0.36964	1.47	0.15439	1.39	0.15390	1.7	0.02400	1	1	0
	GBP5	1.28	0.45032	1.63	0.13124	1.14	0.55480	1.64	0.02940	1	1	0
	IGLC1	4.62	0.03662	2.26	0.08684	2.61	0.01040	1.12	0.74920	1	1	1
	IGKC	9.69	0.10383	-1.12	0.89875	2.55	0.00930	1.45	0.29310	1	0	0
	PMEPA1	1.22	0.72689	3.09	0.04686	1.17	0.66830	2.91	0.00480	1	1	1
	SLC39A8	-1.68	0.12844	-1.33	0.35969	-2.1	0.05000	-1.06	0.86660	1	1	0
	TRMT5	1.36	0.06847	1.56	0.01504	1.1	0.67410	2	0.00370	1	1	1

Supplemental Table S2. Validation of microarray results by RT-PCR for CD14+ samples in longitudinal comparisons. Results from the two types of assays are juxtaposed for the cross-sectional comparison (baseline vs. 2 week or baseline vs. 10 week). Yellow highlights indicate p < .05 for a comparison. In each case, it is specified whether the two assays report the gene changing in the same direction and whether the change is significant at p < .05 for at least one of the time points ("TaqMan validates" column).
