# Supplementary material for: Divergent Gene Activation in Peripheral Blood and Tissues of Patients with Rheumatoid Arthritis, Psoriatic Arthritis and Psoriasis following Infliximab Therapy
Source: PLoS One. 2014 Oct 21;9(10):e110657. doi: 10.1371/journal.pone.0110657 (PMC4204991; doi:10.1371/journal.pone.0110657)
Supplement: Table S3 — Validation of microarray results by RT-PCR for CD14+ samples in cross-sectional comparisons. (PDF) [file pone.0110657.s008.pdf]

CD14+ CROSS SECTIONAL COMPARISON VALIDATION							
**Disease**	**Gene**	**TaqMan**		**Microarray**		**Same Direction**	**TaqMan Validates**
		**FC**	**raw p**	**FC**	**raw p**		
**RA**	CIITA	-4.15032	0.00000	-2.26	0.00000	1	1
	CKAP2	-1.75991	0.00518	-1.87	0.04830	1	1
	FCGR1A	1.64035	0.00247	1.79	0.00000	1	1
	GBP4	-2.45028	0.02526	-1.68	0.00430	1	1
	SLC39A8	2.51470	0.00447	4.28	0.00005	1	1
**PsA**	CKAP2	-1.49542	0.03226	-2.00	0.01760	1	1
	IGKC	-1.53961	0.71550	-2.07	0.02490	1	0
	IGLC1	-1.11803	0.85148	-2.34	0.01720	1	0
	SLC39A8	1.90743	0.03207	1.95	0.03020	1	1
	THBS1	2.82265	0.03939	4.83	0.00003	1	1
**Ps**	SLC39A8	1.80553	0.05640	1.99	0.02280	1	0

Supplemental Table S3. Validation of microarray results by RT-PCR for CD14+ samples in cross-sectional comparisons. Results from the two types of assays are juxtaposed for the cross-sectional comparison (Healthy vs Disease). Yellow highlights indicate $p < .05$ for a comparison. In each case, it is specified whether the two assays report the gene changing in the same direction and whether the change is significant at $p < .05$ (“TaqMan validates” column).
