# Supplementary material for: Divergent Gene Activation in Peripheral Blood and Tissues of Patients with Rheumatoid Arthritis, Psoriatic Arthritis and Psoriasis following Infliximab Therapy
Source: PLoS One. 2014 Oct 21;9(10):e110657. doi: 10.1371/journal.pone.0110657 (PMC4204991; doi:10.1371/journal.pone.0110657)
Supplement: Table S4 — Validation of microarray results by RT-PCR for biopsy samples. (PDF) [file pone.0110657.s009.pdf]

PS BIOPSY COMPARISON VALIDATION														
Group	Gene	Refs	Cross Sectional (L0 vs NL0)						Longitudinal (L2 vs L0)					
			TaqMan		Microarray		Same Direction	TaqMan Validates	TaqMan		Microarray		Same Direction	TaqMan Validates
			FC	raw p	FC	raw p			FC	raw p	FC	raw p		
Significant in Cross-Sectional (L0 vs NL0) Comparison	CASP1		1.56	0.1073	2.21	0.00005	1	0	-1.21	0.1909	-1.77	0.00129	1	0
	CD24		5.69	0.0016	6.53	0.00039	1	1	-2.62	0.0528	-2.60	0.01711	1	0
	CD274		36.90	0.0077	2.58	0.00052	1	1	-6.37	0.0086	-2.07	0.00172	1	1
	ELF3		2.82	0.0006	5.47	0.00018	1	1	-2.75	0.0382	-5.06	0.00949	1	1
	FAM174B		-2.13	0.0674	-2.00	0.00002	1	0	1.66	0.1561	1.46	0.10073	1	1
	FYN	3	-3.10	0.0007	-2.20	0.00041	1	1	1.74	0.0379	1.14	0.40227	1	0
	H19	1	-21.09	0.0606	-4.90	0.00036	1	0	11.04	0.1379	2.77	0.09109	1	1
	IL19		8890.20	0.0017	28.40	0.00047	1	1	-89.16	0.0379	-15.63	0.00042	1	1
	KLK13	1	6.52	0.001	14.68	0.00008	1	1	-3.13	0.0237	-5.56	0.01095	1	1
	KYNU	1	22.08	0.0002	21.53	0.00003	1	1	-2.11	0.0081	-2.59	0.00345	1	1
	LCN2	1	65.47	0.0003	34.39	0.00011	1	1	-6.21	0.0609	-5.11	0.05218	1	0
	LYN		2.09	0.0165	2.61	0.00000	1	1	-1.49	0.0265	-2.10	0.00145	1	1
	OASL	1,2	11.83	0.0002	9.67	0.00037	1	1	-1.73	0.3726	-2.15	0.13663	1	1
	RHCG	1	20.17	0.0047	26.83	0.00028	1	1	-10.15	0.0474	-7.52	0.00891	1	1
	S100A12	1	49.34	0.0003	25.39	0.00017	1	1	-6.62	0.0568	-5.42	0.04693	1	0
	SERPINB1	2	2.55	0.0191	4.18	0.00308	1	1	-3.48	0.0007	-4.09	0.00032	1	1
	SERPINB13	1	5.00	0.0044	4.07	0.00042	1	1	-1.60	0.2514	-1.66	0.09669	1	1
	SORBS1		-6.48	0.0271	-4.37	0.00048	1	1	2.29	0.0878	1.54	0.03455	1	0
	TCN1	1	176.44	0.0001	112.67	0.00012	1	1	-8.56	0.0471	-7.10	0.04933	1	1
	TMPRSS11D	1	346.28	0.0003	37.69	0.00012	1	1	-7.27	0.0503	-5.35	0.02061	1	0
	TRAF3IP2		-1.18	0.1354	-2.02	0.00004	1	0	1.24	0.0869	1.41	0.14352	1	1
	VNN3	1	47.74	< 0.0001	13.72	0.00001	1	1	-3.31	0.0482	-3.25	0.01632	1	1
Not Significant, Highly Expressed in Cross-Sectional	KLK7		1.03	0.918	1.21	0.10525			1.43	0.3529	1.09	0.07745		
	KRT10		-1.54	0.3044	-1.11	0.43191			1.76	0.1104	1.12	0.03005		
Not Significant, Weakly Expressed in Cross-Sectional	BCL2		-5.33	0.0009	-1.82	0.08810			2.38	0.3039	-1.25	0.05565		
	IRF5		-1.45	0.2185	1.08	0.52245			1.27	0.4119	-1.25	0.20227		
Significant in Longitudinal (L2 vs L0) Comparison (and in Figure 5 except for NOS1)	CENPN		5.78	0.0055	2.23	0.00288	1	1	-4.94	0.0025	-2.20	0.00486	1	1
	CKAP2L		1.41	0.2675	1.99	0.02826	1	0	-1.58	0.249	-1.83	0.00051	1	0
	CMAHP		-5.03	0.0017	-2.23	0.00145	1	1	2.16	0.0289	1.95	0.00926	1	1
	GBP1		2.71	0.0375	3.72	0.00240	1	1	-1.83	0.0233	-2.32	0.00825	1	1
	GBP5		4.12	0.0312	3.03	0.00324	1	1	-2.69	0.007	-2.61	0.00279	1	1
	IFNG		17.20	0.0085	2.99	0.02583	1	1	-1.98	0.0315	-2.31	0.00852	1	1
	SLC9A1		-3.10	0.0712	1.87	0.01417	0	0	2.49	0.3967	-1.82	0.00196	0	0
	NOS1		-20.65	0.0777	2.71	0.00044	0	0	33.61	0.0293	2.71	0.00044	1	1
Discussion Genes	1F127		4.15	0.0352	2.85	0.00572	1	1	-1.01	0.9833	-1.01	0.85856	1	1
	AQP9		-2.91	0.0179	-2.08	0.01466	1	1	1.69	0.2978	1.22	0.49397	1	1
	ISG20		1.88	0.0466	3.40	0.00263	1	1	-1.26	0.3547	-2.13	0.00229	1	0
	RAB31		1.87	0.0022	1.67	0.00051	1	1	-1.30	0.1784	-1.29	0.00091	1	0
	SYK		1.14	0.5518	1.78	0.00016	1	0	1.23	0.1478	-1.35	0.04447	0	0
	WNT5A		10.58	0.079	4.38	0.01518	1	0	1.41	0.6389	-1.57	0.11194	0	1

Ps biopsy callout genes

1. Suarez-Farinas et al (2012) Expanding the psoriasis disease profile: interrogation of the skin and serum patients with moderate-to-severe psoriasis. J. Invest. Derm. 132:2552-2564.
2. Yao et al (2008) Type I interferon: potential therapeutic target for psoriasis? PloS One DOI: 10.1371/journal.pone.0002737.
3. Manczinger and Kemeny (2013) Novel factors in the pathogenesis of psoriasis and potential drug candidates are found with systems biology approach. PloS One DOI:10.1371/journal.pone.0080751.

Supplemental Table S4. Validation of microarray results by RT-PCR for biopsy samples. Results from the two types of assays are juxtaposed for the cross-sectional comparison (L0 vs NL0, left) and the longitudinal comparison (L2 vs L0, right). Yellow highlights indicate p < .05 for a comparison. In each case, it is specified whether the two assays report the gene changing in the same direction and whether the change is significant at p < .05 ("TaqMan validates" column). Cells with blue outline are those used to compute "validation rate" in manuscript.
