# Supplementary material for: Divergent Gene Activation in Peripheral Blood and Tissues of Patients with Rheumatoid Arthritis, Psoriatic Arthritis and Psoriasis following Infliximab Therapy
Source: PLoS One. 2014 Oct 21;9(10):e110657. doi: 10.1371/journal.pone.0110657 (PMC4204991; doi:10.1371/journal.pone.0110657)
Supplement: Table S5 — TaqMan primers used for validation. (PDF) [file pone.0110657.s010.pdf]

Gene Symbol	TaqMan® Gene Expression Assay ID
AQP9	Hs01035888_m1
BCL2	Hs00153350_m1
BLNK	Hs00179459_m1
CASP1	Hs00354832_m1
CD24	Hs03044178_g1
CD274	Hs00204257_m1
CENPN	Hs00218401_m1
CIITA	Hs00172094_m1
CKAP2	Hs00217068_m1
CKAP2L	Hs00403991_m1
CMAHP	Hs00186003_m1
ELF3	Hs00963881_m1
FAM174B	Hs01651836_m1
FCGR1A; FCGR1B, FCGR1C	Hs00174081_m1
FYN	Hs00941600_m1
GAPDH	Hs99999905_m1
GBP1	Hs00977005_m1
GBP4	Hs00925073_m1
GBP5	Hs00369472_m1
H19	Hs00262142_g1
IFI27	Hs01086370_m1
IFNG	Hs00989291_m1
IGKC	Hs02384840_gH
IGLC1;IGLL5	Hs00760769_s1
IL19	Hs00604657_m1
IRF5	Hs00158114_m1
ISG20	Hs00158122_m1
KLK13	Hs01087307_m1
KLK7	Hs00192503_m1
KRT10	Hs01043114_g1
KYNU	Hs01114093_m1
LCN2	Hs01008571_m1
LYN	Hs00176719_m1
NOS1	Hs00167223_m1
OASL	Hs00388714_m1
PMEPA1	Hs00375306_m1
RAB31	Hs00199313_m1
RHCG	Hs00170975_m1
S100A12	Hs00194525_m1
SERPINB1	Hs00961948_m1
SERPINB13	Hs00202640_m1
SLC39A8	Hs00223357_m1
SLC9A1	Hs00300047_m1
SORBS1	Hs00908953_m1
SYK	Hs00895377_m1
TCN1	Hs01055542_m1
THBS1	Hs00962908_m1
TMPRSS11D	Hs00975370_m1
TRAF3IP2	Hs00974570_m1
TRMT5	Hs00418256_m1
VNN3	Hs00218179_m1
WNT5A	Hs00998537_m1

Life Technologies, Grand Island NY, www.lifetechnologies.com

Table S5. TaqMan primers used for validation.
